# Rectal stump management in paediatric ulcerative colitis—An international survey for the European Society for Pediatric Gastroenterology, Hepatology, and Nutrition PORTO Inflammatory Bowel Disease group study

**DOI:** 10.1002/jpr3.70198

**Published:** 2026-07-13

**Authors:** Beth Gordon, Sarah Cooper, Billy Bourke, Séamus Hussey

**Affiliations:** ^1^ National Centre for Gastroenterology, Hepatology and Nutrition, Children's Health Ireland Dublin Ireland; ^2^ School of Medicine University College Dublin Dublin Ireland; ^3^ DOCHAS Study, Children's Health Ireland Dublin Ireland

**Keywords:** colectomy, inflammatory bowel disease, questionnaire

## Abstract

**Objective:**

To evaluate current international clinical practice in the assessment and management of rectal stump disease activity following colectomy in paediatric ulcerative colitis (UC).

**Methods:**

A web‐based survey comprising 18 questions was distributed to paediatric gastroenterologists via European Porto inflammatory bowel disease (IBD) interest group and North America and the United Kingdom pediatric inflammatory bowel disease (PIBD) network groups over 6 months. The questionnaire recorded centre demographics, colectomy practices, rectal stump assessment methods, and management approaches. Responses were analysed using descriptive statistics with subgroup analysis by continent and centre size.

**Results:**

Eighty paediatric gastroenterologists from 24 countries completed the survey. Most centres (*n* = 62, 78%) reported fewer than five colectomies annually, with 59% indicating that all patients retained a rectal stump for at least 3 months post‐colectomy. Only 11% of centres had a formal protocol for rectal stump management. Clinical assessment tools varied, with 39% using none, and the remainder favouring Pediatric Ulcerative Colitis Activity Index (PUCAI) or Physician's Global Assessment. Frank rectal bleeding and painful rectal discharge were rated as the most significant symptoms. Endoscopy and histology were the most common investigations. Rectal therapy was the preferred first‐line treatment for rectal stump disease recurrence, followed by systemic therapy and observation. There was wide variation in estimates of rectal disease activity at 6 months post‐colectomy.

**Conclusions:**

There is considerable international variation and lack of standardisation in the management of rectal stump disease in paediatric UC. These findings underscore the need for evidence‐based guidelines and collaborative research to optimise care for this understudied population.

## Abstract (highlights)

**What is Known:**

- The interim time period from colectomy to further restorative surgery varies considerably across centres according to access to surgical expertise.
- During this interval, patients can present with a variety of symptoms from the residual rectal stump requiring further medical management.
- There is a large paucity of data in the literature regarding how to best assess, investigate and medically manage the rectal stump.

**What is New:**

- This survey demonstrates substantial heterogeneity in clinical practice regarding rectal stump disease recurrence across a large cohort of international experts.
- This survey outlines the critical need for targeted research into treatment, management, and outcomes of paediatric patients with residual rectal stumps.

## 1 INTRODUCTION

Ulcerative colitis (UC) in childhood is associated with a higher risk of developing acute severe colitis and colectomy compared to the adult population.^1^ Colectomy indications include acute colonic perforation, toxic megacolon, medically unresponsive acute severe colitis (ASC), and is the next‐line treatment following unsuccessful medical therapy for chronically active refractory disease.^2^, ^3^ The typical surgical approach in paediatric UC involves a staged procedure, with the initial operation consisting of colonic resection (subtotal colectomy) and formation of an end‐ileostomy, leaving the oversewn rectal stump in situ.^4^

The timing of the subsequent 1 or 2 restorative surgical stages varies internationally, due to patient and clinician preferences or access to specialised surgical expertise. Current recommendations advise that restorative ileal pouch‐anal anastomosis (IPAA) surgery is performed by experienced surgeons in high‐volume centres that perform at least 10 pouches/year, due to improved pouch outcomes and lower pouchitis rates.^2^ During this interim period, active disease in the residual rectal stump has the potential to cause clinical symptoms.

Despite potentially significant symptoms, there is a paucity of literature regarding residual rectal disease activity during the interval between colectomy and restorative surgery. The clinical assessment, medical management, and outcomes of the rectal stump have not been adequately described, particularly in children. Whilst clinicians counsel patients that post‐operative symptoms may arise, the likelihood and prevalence of such manifestations have been poorly documented.

This study aims to characterise current international clinical practice patterns in the management of rectal stump disease following colectomy in paediatric UC patients, identifying areas of consensus and variation to inform future research directions and clinical guideline development.

## 2 METHODS

### 2.1 Ethics statement

Participation was voluntary and anonymous. Ethical approval was obtained from Children's Health Ireland, Dublin (REC 316‐23).

### 2.2 Design

A cross‐sectional, web‐based survey was designed to assess current clinical practice in the management of rectal stump disease following colectomy in paediatric UC (Supporting Information S1: Table S1). The survey instrument was developed by the study authors and reviewed by experienced paediatric gastroenterologists within the Porto Group of European Society for Pediatric Gastroenterology, Hepatology, and Nutrition (ESPGHAN) for content validity. The English language survey comprised 18 questions, including a combination of open, closed, and ranking formats, and was distributed via the SurveyMonkey platform. The survey was available for a 6‐month period (November 2023 to April 2024).

The survey was structured into four domains: (1) centre demographics, (2) colectomy practice, (3) rectal stump assessment and (4) rectal stump management. Respondents were asked about annual colectomy numbers at their centre, the proportion of patients with a rectal stump in situ, the existence of local protocols, assessment tools, symptom expectations, investigation preferences, and treatment approaches.

### 2.3 Recruitment

Eligible participants were paediatric gastroenterologists involved in the care of children with inflammatory bowel disease (IBD) and experience of managing UC requiring colectomy. The survey was disseminated through established networks including the ESPGHAN Porto IBD Special Interest Group, and paediatric gastroenterology society groups and networks in Britain, Europe and North America.

### 2.4 Statistical analysis

Responses were analysed using descriptive statistics. Subgroup analyses were conducted by continent and centre size, with centres classified according to the total paediatric IBD patient cohort as small (<100 patients), moderate (100–300), large (301–500), or very large centres (>500). Ranking questions, used to determine the relative importance of clinical symptoms and management options, utilised weighted scoring systems, with first‐position responses receiving the highest weighting. The weighted values were adjusted to match the number of choices selected and the scores were calculated on that basis. Free‐text responses were reviewed for additional insights.

## 3 RESULTS

**Figure 1:**
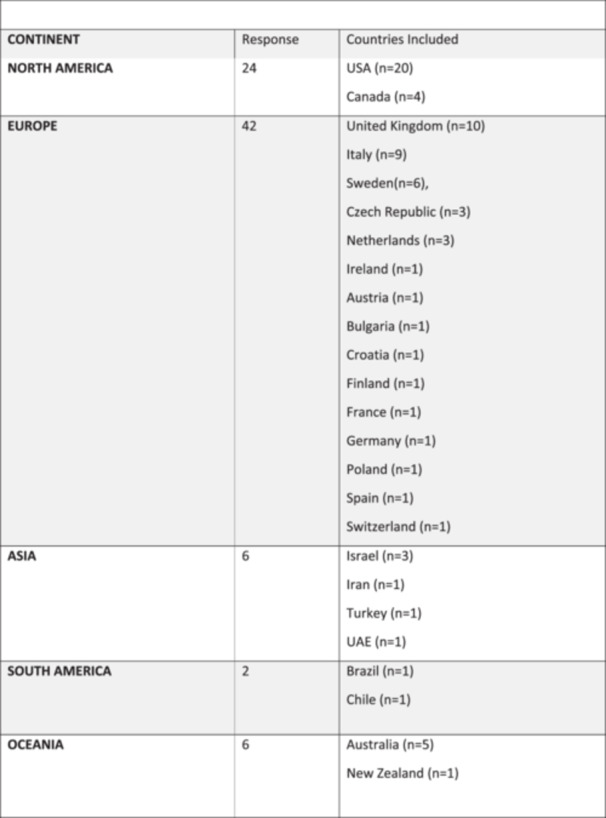
Breakdown of responses by continent.

A total of 80 paediatric gastroenterologists from 24 countries across Europe, North America, South America and Oceania completed the survey (Figure 1). Centres were categorised by volume size according to total PIBD patient cohort. Respondents were distributed across centres of varying IBD patient volumes: 11 (14%) from small, 34 (42%) from moderate, 20 (25%) from large and 15 (19%) from very large centres. Despite variation in centre sizes, an annual diagnosis rate of approximately 20–40 new cases of UC was the predominant response (Table 1).

**Table 1:** New diagnosis UC per year categorised by centre size.

New diagnosis UC/year	Small centres (PIBD population <100)	Moderate centre (PIBD population 100–300)	Large centre (PIBD population 301–500)	Very large centre (PIBD population >500)
<20	8	16	2	0
20–40	3	16	12	7
41–60	0	0	4	5
>60	0	1	2	3

Abbreviations: PIBD, Pediatric inflammatory bowel disease; UC, ulcerative colitis.

### 3.1 Colectomy practices

Respondents were asked about colectomy practises at their centres, with 79% (*n* = 62) reporting less than five colectomies per annum in the preceding 10 years. Following colectomy, 59% (*n* = 39) of respondents report that all patients at their centre have a rectal stump in situ for at least 3 months, and a further 30% reported that a majority of patients have a rectal stump. Only 5% (*n* = 3) of respondents reported no patients retain their rectal stump for at least 3 months, suggesting either single‐stage IPAA practice or a short interval (<3 months) 2‐stage IPAA local practice.

Analysis by region revealed that patients in the United Kingdom and Asia were more likely than those in Europe and North America to retain their rectal stump for longer periods with 71% of UK and Asian respondents reported managing patients with a rectal stump in situ for ≥3 months, compared to 50% of respondents from Europe and North America.

Despite the high proportion of patients with residual rectal stumps, 89% (*n* = 58) of respondents indicated that no formal protocol or treatment guideline exists at their centre for managing rectal disease recurrence. Those centres with written management guidelines were located in the United Kingdom, Sweden and Italy, and all were large‐volume centres.

### 3.2 Rectal stump assessment

Significant heterogeneity was observed in rectal stump clinical assessment approaches. Among respondents, 39% (*n* = 26) do not use a specific assessment tool, whilst 27% (*n* = 18) employed the Paediatric Ulcerative Colitis Activity Index (PUCAI) and 24% (*n* = 16) used a Physician Global Assessment (PGA) approach.

Regarding clinician's post‐operative expectations, 63% (*n* = 41) anticipated mild to tolerable symptoms occurring intermittently until stoma reversal, while 19% (*n* = 12) expected symptoms to resolve within 6 weeks of colectomy. Only 11% (*n* = 7) anticipated no symptom resolution until further surgery, and 4% (*n* = 3) expected total symptom resolution by hospital discharge.

**Figure 2:**
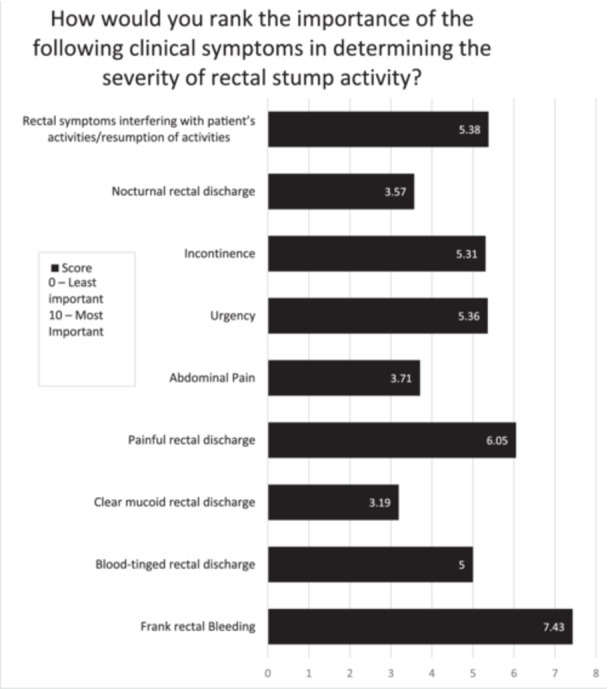
Clinical symptoms in determining severity ranking. Weighted Scoring System: Participants ranked choices in order of likelihood. 1st choice‐position responses receiving the highest weight. The weighted values were adjusted to match the number of choices selected and the scores were calculated on that basis. 0—Least important, 10—Most important.

When ranking clinical symptoms for determining rectal stump activity severity, frank rectal bleeding received the highest weighted score, followed by painful rectal discharge. When asked to consider the patient and carer perspective, frank rectal bleeding remained the highest‐ranking concern, followed by rectal symptoms interfering with daily activities (Figure 2).

Raised markers of inflammation (C‐reactive protein/erythrocyte sedimentation rate), iron deficiency anaemia and rectal stricture formation were identified as the most important concerning clinical features by respondents.

Regarding routine investigations, 90% (*n* = 50) of respondents perform endoscopy and histological assessment of symptomatic rectal stumps, 81% (*n* = 44) routinely employ blood indices and 60% (*n* = 33) utilise calprotectin levels on rectal stump output.

**Figure 3:**
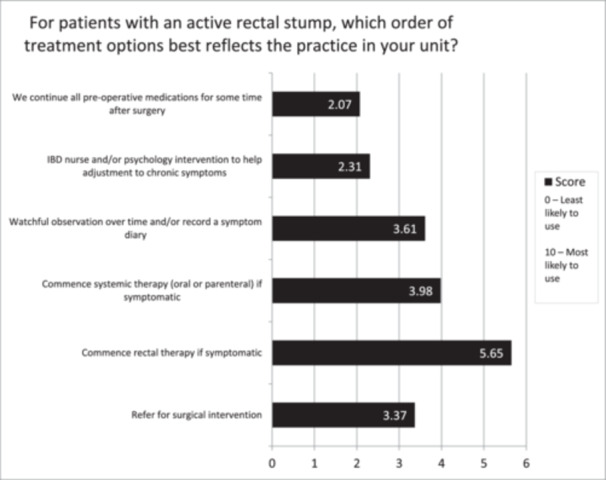
Treatment preferences for rectal stump disease recurrence ranking. Weighted Scoring System: Participants ranked choices in order of likelihood. 1st choice‐position responses receiving the highest weight. The weighted values were adjusted to match the number of choices selected and the scores were calculated on that basis. 0—Least likely to use, 10—Most likely to use.

When ranking treatment preferences for rectal stump disease recurrence, rectal therapy emerged as the highest‐ranking option, with 72% of respondents rating it as their first preference, aligning with current European Crohn's and Colitis Organisation (ECCO)/ESPGHAN guidelines. However, which specific rectal treatment used was not recorded. Systemic therapy ranked second, followed by watchful observation (Figure 3).

For determining remission status in patients with previously active rectal stump disease, resolution of all symptoms received the highest weighted score (69%), while 15% of respondents ranked normalised endoscopic appearance to determine remission status.

Estimates of the proportion of patients with minimal rectal activity at 6 months post‐colectomy varied considerably: 31% of respondents estimated 35%–50% of patients, 29% estimated 20%–35%, 20% estimated <20% and 18% estimated >50%.

## 4 DISCUSSION

This study provides the first comprehensive insight into worldwide clinical practice regarding paediatric rectal stump management following colectomy for UC. The literature addressing rectal stump outcomes and management remains limited and primarily focuses on adult populations, endoscopic surveillance for dysplasia and surgical management outcomes. Medical management of symptomatic rectal stumps during the interval between colectomy and restorative surgery has not been well‐documented, particularly in children.

Previous studies demonstrated significant variation in timing between colectomy and restorative surgery. The ESPGHAN Porto IBD Group study of IPAA outcomes reported a median interval time of 6 months (range 3–12 months) from colectomy to restoring ileal continuity in 129 patients, while a single centre study reported a median interval of 1.2 years (0.7–4.1 years) to pouch surgery.^5^, ^6^ The latter group specifically deferred pouch surgery until later teenage years. Our survey affirms that for many centres paediatric patients wait until transition to adult services to access this expertise. These extended intervals create a substantial opportunity for rectal stump activity and highlights the need for optimised care strategies.

The heterogeneity in clinical assessment tools and management approaches revealed by our survey reflects the absence of specific literature and evidence‐based guidelines. Certain clinical factors have been associated with pouchitis for example, including extraintestinal manifestations and possibly high PUCAI scores at diagnosis.^7^ However, no such factors are known or have been studied systematically regarding rectal stump disease. The preference for rectal therapy aligns with current ECCO/ESPGHAN recommendations, though variable implementation by centres also reflects a lack of standardised approaches in real‐world settings. The wide range of estimates regarding disease activity at 6 months post‐colectomy (from <20% to >50%) further highlights limited awareness and the need for systematic data collection and analysis in this small but important patient demographic.

The STRIDE‐II (Selecting Therapeutic Targets in Inflammatory bowel disease) guidelines emphasise quality of life as a key treatment goal in paediatric IBD care.^8^ Our survey confirms that clinicians recognise the significant impact of rectal stump symptoms on patients’ daily activities, highlighting the clinical relevance of this condition for patients and care givers.

Whether patients’ subjective clinical stump symptoms correlate with objective laboratory or other markers remains to be established. Furthermore, the potential impact of optimal endoscopic healing on subsequent surgical or pouch outcomes requires investigation. Rectal mesalazine was previously shown to be effective (clinically, endoscopically, histologically) in adults with post‐IPAA ‘cuffitis’, suggesting potential therapeutic applications in the pre‐IPAA period also.^9^ However, carefully designed studies are needed to fill the current knowledge gap in rectal stump therapy.

This survey represents the first examination of a previously unstudied topic but has several limitations. Responses were based on personal experience rather than objective data, potentially introducing bias towards motivated practitioners. The survey questions were not compulsory so there were discrepancies in the response rate of certain questions, especially the ranking questions. Participants were asked to report their country of practice rather than treating centre (to maintain anonymity), so it was not possible to determine the true geographic spread of respondents from the same country. The predominance of respondents from developed countries limits generalisability to diverse healthcare settings, although it is possible that access to IPAA may be more challenging low resource settings. However, the aim of the survey was to provide a broad assessment of clinical experience rather than precise epidemiological data, making it suitable for this scoping review of international practice.

## 5 CONCLUSION

This international survey reveals substantial heterogeneity in clinical practice for managing rectal stump disease in paediatric UC. The findings underscore the critical need for targeted research into treatment, management, and outcomes of paediatric patients with residual rectal stumps. A multicentre collaborative effort is needed to generate sufficient data for future evidence‐based guidelines and improve consistency in care during the crucial interval between colectomy and restorative surgery.

Future research should focus on establishing standardised assessment tools, investigating the correlation between clinical symptoms and objective inflammatory markers, and evaluating whether it impacts outcomes of pouch surgery, including pouchitis and cuffitis.

## CONFLICT OF INTEREST STATEMENT

Séamus Hussey: Unrelated grant funding from Janssen. The remaining authors declare no conflicts of interest.

## Supporting information

Supporting File 1
