# Supplementary material for: Rectal stump management in paediatric ulcerative colitis—An international survey for the European Society for Pediatric Gastroenterology, Hepatology, and Nutrition PORTO Inflammatory Bowel Disease group study
Source: JPGN Rep. 2026 Jul 13:10.1002/jpr3.70198. Online ahead of print. doi: 10.1002/jpr3.70198 (PMC13394627; doi:10.1002/jpr3.70198)
Supplement: Supplementary file 1 — Supporting File 1 [file JPR3-9999-0-s001.docx]

**Supplementary Data Table 1 – Survey Questions and Answers**

|  | Question | Answer |
| --- | --- | --- |
| 1 | In which country do you practise? | Freetext |
| 2 | Approximately how many patients with PIBD are treated in your centre? | - 0-100 (Small) - 101-300 (Moderate) - 301-500 (Large) - >500 (Very large) |
| 3 | Approximately how many new diagnoses of UC are made annually? | - <20 - 21-40 - 41-60 - >61 |
| 4 | Over the past 10 years, how many patients with UC undergo colectomy each year, on average? | - <5 - 5-10 - >10 |
| 5 | Following subtotal colectomy for UC, do your patients have an oversewn rectal cuff for a minimum of three months? | - Yes – All do - Yes- majority do - Yes – minority do - No - none - Other approach used- please specify:- Freetext |
| 6 | What assessment tool do you use to assess rectal cuff activity? | - None specifically - PUCAI - PGA - Other - please specify: Freetext |
| 7 | Do you have a protocol or treatment guideline for managing rectal cuff disease? | Yes  No |
| 8 | Which advice best reflects the usual practice approach for patients with an oversewn rectal cuff post-colectomy? | - Expect all/almost all symptoms to resolve by the time of hospital discharge - Expect all symptoms to steadily resolve within 6 weeks of colectomy - Expect mild/tolerable symptoms to occur intermittently until stoma-reversal surgery occurs - Have no/limited expectation that symptoms will resolve fully until stoma-reversal surgery occurs - None of our patients have a residual rectal cuff as defined above |
| 9 | How would ***you*** rank the importance of the following clinical symptoms in determining the severity of rectal cuff activity? | - - Frank rectal bleeding   - Blood-tinged rectal discharge   - Clear mucoid rectal discharge   - Painful rectal discharge   - Abdominal Pain   - Urgency   - Incontinence   - Nocturnal rectal discharge   - Rectal symptoms interfering with patient’s activities/resumption of activities   - Symptom frequency   - Other (please state) |
| 10 | How do you think ***patients and/or carers*** would rank the following clinical symptoms in determining the severity of rectal cuff activity? | - Frank rectal bleeding - Blood-tinged rectal discharge - Clear mucoid rectal discharge - Painful rectal discharge - Abdominal Pain - Urgency - Incontinence - Nocturnal rectal discharge - Rectal symptoms interfering with patient’s activities/resumption of activities - Other (please state) |
| 11 | How would you rank the importance of the following features or signs in determining the severity of rectal cuff activity? | - Extraintestinal manifestations of IBD (new/ongoing/recurrence) - Iron-deficient anaemia - Elevated blood inflammatory markers (ESR/CRP) - High white cell count - High platelet count - Hypoalbuminaemia - Oligomenorrhoea/amenorrhoea - Abnormal findings on perianal inspection - Rectal stricture formation - Other (please state): |
| 12 | What investigations do you routinely undertake in patients reporting rectal cuff disease activity? (Can check multiple boxes) | Blood laboratory indices (e.g. haematology/biochemistry)  Rectal calprotectin level  Rectal FIT/occult blood testing  Endoscopy +/- histology of rectal cuff  Clinical assessment only (e.g. symptom profile or symptom diary)  Culture/ microbial analysis of rectal discharge  Bowel/abdominal ultrasound  MRI (pelvis/rectum/enterogram)  CT  Transrectal ultrasound  Examination under anaesthesia (without endoscopy)  Other (state): |
| 13 | For patients with an active rectal cuff, which order of treatment options best reflects the practice in *your unit*? | Refer for surgical intervention  Commence rectal therapy if symptomatic  Commence systemic therapy (oral or parenteral) if symptomatic  Watchful observation over time and/or record a symptom diary  IBD nurse and/or psychology intervention to help adjustment to chronic symptoms  We continue all pre-operative medications for some time after surgery |
| 14 | Which order of options best reflects your practice in determining remission status in patients with a previously active rectal cuff disease (e.g. following a treatment intervention)? | Resolution of all symptoms  Return to baseline symptom profile  Normalised laboratory parameters  Normalised endoscopic appearance  Normalised histology  Normal radiology  Normalised rectal calprotectin  Remission per PUCAI score definition |
| 15 | In your estimation of your centre’s experience, what proportion of patients with a residual oversewn rectal cuff are asymptomatic or have minimal rectal activity by 6 months post-colectomy? | 0-20%  21-33%  34-50%  >51% |
| 16 | In your opinion, is it worthwhile undertaking a research study of post-colectomy rectal cuff disease activity? | Yes  No |
| 17 | Would you be interested in participating in a study focused on rectal cuff disease following colectomy? | Yes  No |
